# The Therapeutic Potential of Ethnomedicinally Important Anatolian Thyme Species: A Phytochemical and Biological Assessment

**DOI:** 10.3389/fphar.2022.923063

**Published:** 2022-06-09

**Authors:** Esra Eroglu Ozkan, Ezgi Ersoy, Yeter Yesil Canturk, Emel Mataraci Kara, Ercan Cinar, Hasan Sahin, Selim Karahan, Kubra Karaca Sancaktepe, Mustafa Abdullah Yilmaz, Mehmet Boga

**Affiliations:** ^1^ Department of Pharmacognosy, Faculty of Pharmacy, Istanbul University, Istanbul, Turkey; ^2^ Department of Pharmacognosy, Faculty of Pharmacy, Biruni University, Istanbul, Turkey; ^3^ Department of Pharmaceutical Botany, Faculty of Pharmacy, Istanbul University, Istanbul, Turkey; ^4^ Department of Pharmaceutical Microbiology, Faculty of Pharmacy, Istanbul University, Istanbul, Turkey; ^5^ Department of Nursing, School of Health Sciences, Batman University, Batman, Turkey; ^6^ Department of Pharmacognosy, Faculty of Pharmacy, Dicle University, Diyarbakır, Turkey; ^7^ Department of Laboratory Animals, Faculty of Veterinary, Dicle University, Diyarbakır, Turkey; ^8^ Dicle University Health Sciences Application and Research Center (DÜSAM), Diyarbakır, Turkey; ^9^ Department of Analytical Chemistry, Faculty of Pharmacy, Dicle University, Diyarbakır, Turkey

**Keywords:** Thyme, *Thymus* sp., Anatolian, LC-MS/MS, antioxidant, anti-cholinesterase activity, cytotoxicity, *Candida tropicalis*

## Abstract

Thyme has been used for various therapeutic purposes in many different cultures, which makes it one of the most riveting medicinal plants throughout history. From its beneficial effects on the respiratory tract or the gastrointestinal system, to its unique skin-related activities, the investigation of the medicinal properties of thyme has always been an alluring topic for researchers aiming to develop conventional medications from this traditional herb. With an incentive to contribute to the extensive thyme research, three *Thymus* L. species namely *Thymus cariensis* Hub-Mor. & Jalas (endemic), *Thymus praceox* subsp. *grossheimii* (Ronniger) Jalas, and *Thymus pubescens* Boiss. et Kotschy ex Celak from Turkey were deeply investigated within this study. The analysis of the phytochemical constituents of the extracts was conducted by LC-MS/MS. 12 biologically important secondary metabolites (*p*-coumaric acid, caffeic acid, salicylic acid, quinic acid, fumaric acid, vanillin, malic acid, rutin, apigenin, naringenin, and nicotiflorin) were detected in all extracts. Their total phenolic and flavonoid contents were calculated (11.15 ± 0.17—61.12 ± 2.59 μg PEs/mg extract, 2.53 ± 0.04—40.28 ± 0.92 μg QEs/mg extract, respectively), and the antioxidant potential of the extracts was evaluated by DPPH and ABTS radical scavenging and CUPRAC activity methods, accordingly, the extracts were shown to possess significant antioxidant activity. Among them, *Thymus cariensis* Hub-Mor. & Jalas was the most active with IC_50_ values of 34.97 ± 1.00 μg/ml and 9.98 ± 0.04 μg/ml regarding the DPPH and ABTS radical scavenging assays, respectively, and an A_0.5_ value of 5.80 ± 0.02 μg/ml according to CUPRAC activity method. Their anticholinesterase, antityrosinase, and antiurease activities were also tested, *Thymus cariensis* Hub-Mor. & Jalas (35.61 ± 1.20%) and *Thymus pubescens* Boiss. et Kotschy ex Celak aerial part extract (33.49 ± 1.39%) exhibited moderate antibutyrylcholinesterase activity at 200 μg/ml concentration. The results of the cell viability assay indicated that the extracts demonstrated moderate-to-low cytotoxicity on A498 human renal cell lines. Furthermore, all studied extracts exerted noteworthy antimicrobial activity, especially against *Candida tropicalis* (MIC values: 19.53—78.12 μg/ml). The presented data substantiates the use of thyme extracts as therapeutic agents in both ethnomedicine and conventional therapies.

## 1 Introduction

Using medicinal plants for therapeutic purposes dates back to the Neanderthal period according to the archaeological evidence. The discovery of the 65,000-year-old pollen fossils found in Shanidar Caves (Northern Iraq), has been considered the proof of the long tradition of using particular medicinal plants for the treatment of diseases. Another remarkable example from the Neolithic period is the discovery of the famous “Ice Man” Otzi, who was later diagnosed with whipworm infection, and residue of plants with acaricidal activity was found among his possessions (Sendker and Sheridan, 2017). It is also known that the first humans were also the first cultivators of plants, not only aiming to provide themselves with the food they needed, but also the medicinal drugs for their well-being (Bonini et al., 2018).

Along with this, from a historical perspective, it is known that the most important drugs that are being commonly used today have originated from plants. Although the extraordinary developments in chemistry led to synthetic drugs superseding the natural compounds in the 20th century, in today’s world, the pharmaceutical companies and academia tend to be less enamored of single synthetic compounds as magic bullet cures due to the high costs, and logical toxicity concerns (Stahl-Biskup and Sáez, 2002). In addition to this, the increasing demand for “more natural, effective, and safer” therapeutic agents by the public has made the herbal market evolve dynamically worldwide. Resulting of this, more and more natural supplements, herbal medicines, or conventional drugs that contain plant-derived compounds can be seen on the shelves of pharmacies, and pharmacognosy, which is the multidisciplinary science of nature-derived pharmaceuticals, is becoming inevitably more popular among researchers.

Since the year 2,000, the ethnopharmacological research has been more diversified also more focused on the medicinal plants that are used as food, as well. *Thymus* species are unquestionably among these plants (Yeung et al., 2018). “Thyme” is the commonly used name for the members of the *Thymus* L. species. The great history of thyme starts with its traditional use supported by only empirical results and debatable observations but continues with scientific data provided by numerous studies including clinical trials. These developments bring about the change of thyme from being a traditional plant and a taste enhancer only, to a serious drug in evidence-based phytotherapy (Zarzuelo and Crespo, 2002). Thyme extracts and oils are the ingredients of almost every herbal product that is used for respiratory system diseases, making thyme-containing products among the most selling herbal medications in many countries. Parenthetically, the high amount of essential oil-producing of *Thymus* species is explained by their adaptive characteristics for dry climate conditions. Owing to their unique leave shapes with dense hairs on them, thymes are known to be quite resistant, and they can survive cold temperatures and aridness (Morales, 2002). Even when they have to face challenging environmental conditions such as drought stress, they were shown to have the ability to develop adaptation to these conditions by activating their stress response and evolving their phytochemistry accordingly (Mahdavi et al., 2020; Ashrafi et al., 2022). Over and above, the members of the genus *Thymus* have significant variations in their morphological characteristics that lead them to be evaluated under a great number of species mostly distributed in the Mediterranean region. In Turkey for instance, with the newly identified species, namely *T. baseri* Öztürk, Yaylacı, Koyuncu & Ocak, the number of *Thymus* species increased to 39 (Ozturk et al., 2022).

The wide medical applications of *Thymus* species are certainly worth mentioning. As a case in point, in Germany, the syrup made with thyme is a commonly prescribed medication by pediatrists for the respiratory problems of children. Thyme oil is known as the favorite aromatherapy oil for respiratory tract diseases (Schulz et al., 2004). As it is used the most commonly for therapeutic purposes, not only for respiratory system-related problems but also for further important diseases, the scientifically proved pharmacological activities of *Thymus* species are countless. Speaking of which, *T. dreatensis* Batt. whole plant extract was shown to demonstrate anticoagulant and antihyperlipidemic activity. *T. serpyllum* L. extract was shown to possess an antihypertensive effect (Michel et al., 2020). *T. vulgaris* L. exerted anti-tumor activity on various cell lines by reducing the tumor volume decreasing cell proliferation and inducing apoptosis (Choudhari et al., 2020). Furthermore, *T. vulgaris* L. is among the most studied species in terms of antibacterial activity, generally resulting in extraordinarily strong activity results (Chassagne et al., 2021). *T. serrulatus* Hochst. ex Benth. aqueous extract was shown to have significant antihyperglycemic activity (Haile et al., 2021).

The diverse pharmacological activities of *Thymus* L. species are attributed to their unique chemical characterization. Although the research has been mostly conducted on the essential oil compositions and their activities, recently the chemical profiling of different *Thymus* extracts prepared with different solvents is being investigated more deeply. Nonvolatile secondary metabolites of thyme include a variety of phenolic compounds, terpenoids, and phytosterols. Speaking of the phenolics, apigenin, naringenin, and their derivatives are usually detected in different *Thymus* extracts. Phenolic acids including rosmarinic acid and caffeic acid, and benzoic acid derivatives are also usually present in the extracts (Li et al., 2019).

It can be observed that, as the research on *Thymus* species continues, the status of these plants has moved from being only some traditional plants collected from some herb collectors without meeting any quality criteria to a rational drug fulfilling the safety and efficacy requirements. On that account, the use of a few *Thymus* species was approved by Commission E monographs, ESCOP monographs, and WHO monographs (Stahl-Biskup, 2002). That being the case, in efforts to shed more light on the properties of these species, further studies are crucial. With this impetus, this study investigates the chemical compositions and biological activities of *Thymus cariensis* Hub-Mor. & Jalas, *Thymus praceox* subsp. *grossheimii* (Ronniger) Jalas and *Thymus pubescens* Boiss. et Kotschy ex Celak were collected from different sites in Turkey. Their secondary metabolites were determined by LC-MS/MS, their total phenolic and flavonoid contents were calculated, and their antioxidant, anticholinesterase, antityrosinase, antiurease, anticancer, and antimicrobial activities were assessed. Towards a new understanding of the medicinal potential of *Thymus* species, findings of the current study may be contributing.

## 2 Materials and Methods

### 2.1 Plant Material

*T. cariensis* Hub-Mor. & Jalas was picked up from Koycegiz—Mugla, *T. praceox* subsp. *grossheimii* (Ronniger) Jalas was collected from Savsat—Artvin, and *T. pubescens* Boiss. et Kotschy ex Celak was picked up from Kars, Turkey, in June 2014. The scientific names of the plant taxa were checked and controlled according to the checklist of the flora of Turkey (Güner et al., 2012) and The Plant List website (The Plant List, 2013). The samples were identified by Assoc. Prof. Dr. Yeter Yesil Canturk. Voucher herbarium specimens (*T. cariensis* Hub-Mor. & Jalas: 116046, *T. praceox* subsp. *grossheimii* (Ronniger) Jalas: 116173, and *T. pubescens*: 117326) are preserved in the Istanbul University Faculty of Pharmacy Herbarium (ISTE). All details about the plant samples can be seen in Table 1.

**TABLE 1:** Plant names, collection details, herbarium numbers, and extract yield of the studied *Thymus* extracts.

Plant names—codes	Extract yield (%)	Collection location	Collection time	Herbarium number
*Thymus cariensis* (Tc)	5.20	Köyceğiz—Muğla	12.06.2014	116046
*Thymus praceox* subsp. *grossheimi* aerial part (TprA) *Thymus praceox* subsp. *grossheimi* root part (TprR)	5.55 1.28	Şavşat-Artvin	15.06.2014	116173
*Thymus pubescens* aerial part (TpuA) *Thymus pubescens root* part (TpuR)	2.86 1.80	Kars	27.06.2014	117326

### 2.2 Extraction

The whole plant including roots (10 g) was macerated in ethanol (100 ml) for 24 h at room temperature and then filtrated through Whatman filter paper (No. 1). The procedure was repeated twice more, and combined filtrates were concentrated by using a rotary evaporator at 40°C. The extract was stored at −20°C until the evaluation.

### 2.3 LC-MS/MS Analysis

A previously validated method (Yilmaz et al., 2018) was carried out to identify the LC-MS/MS analysis of the studied ethanol extracts. 37 standard phenolic compounds and a Nexera model UHPLC (Shimadzu) coupled to a tandem MS instrument were used to determine the chemical composition of the extract. Shimadzu Lab Solutions software was used to process the gathered data from LC-ESI-MS/MS.

### 2.4 Total Phenolic and Flavonoid Contents

The total phenolic content of the extract was determined according to the previously used method by Boga et al. (2016), which is a modified procedure described by Slinkard and Singleton (1977) for the first time. The results were calculated by using followed equation; Absorbance = 0.0409 pyrocatechol (μg) + 0.0495 (*R* ^2^ = 0.9975) and given as micrograms of pyrocatechol equivalents (PEs). The total flavonoid content of the extracts was measured by using the method designed by Moreno et al. (2000), and the results here were expressed as micrograms of quercetin equivalents (QEs). The following equation was used to calculate the total flavonoid contents of the extract; Absorbance = 0.0347 quercetin (μg) + 0.1174 (*R* ^2^ = 0.9992).

### 2.5 Antioxidant Activities

DPPH free radical scavenging, ABTS cation radical scavenging, and CUPRAC activity methods were used to investigate the antioxidant activities of the extract. A procedure described by Blois, 1958 was developed and applied to identify the DPPH free radical scavenging potential of the extract. ABTS cation radical decolorization activity of the extract was conducted by using the method designed by Re et al. (1999). The cupric reducing antioxidant capacity (CUPRAC) of the extract was determined using the method summarized by Apak et al. (2004).

### 2.6 Enzyme Inhibition Activity Assays

A method designed by Ellman et al. (1961) was performed to determine the acetylcholinesterase and butyrylcholinesterase inhibitory activity of the extracts. To determine the tyrosinase and urease inhibition activities of the studied extracts, the same methods were used from previously published papers (Ersoy et al., 2019; Ersoy et al., 2020).

### 2.7 Antimicrobial Activities

Antimicrobial activities of the studied extracts and standard compounds against the strains were carried out using the micro broth dilution method as described by the Clinical and Laboratory Standards Institute (1997, 2006, and 2010). The pathogenic strains are namely *Pseudomonas aeruginosa*, *Escherichia coli*, *Klebsiella pneumoniae*, *Enterococcus faecalis*, *Proteus mirabilis*, *Staphylococcus epidermidis*, *Staphylococcus aureus*, *Candida albicans*, *Candida tropicalis*, and *Candida parapsilosis.* Minimum inhibitory concentrations (MICs) of the extract and the standard compounds were determined by the micro broth dilution technique as described by the CLSI (1997, 2006, and 2010). Cefuroxime-sodium, cefuroxime, ceftazidime, amikacin, amphotericin B, and clotrimazole were used as standard antibacterial and antifungal agents.

### 2.8 Cytotoxic Activities

#### 2.8.1 Cytotoxicity Assay on Renal and Colon Cell Lines

A two-day assay was used to determine the cytotoxic potential of the extracts. XTT bioassay is an *in vitro* antitumor colorimetric assay developed by the Molecular Targets Program (MTP), Assay Development and Screening Section, National Cancer Institute (NCI). The renal cancer cell lines (UO-31 and A498) and the colon cancer cell lines (COLO205 and KM12) were used in the assay. All details of the assay were performed according to Eroglu Ozkan et al., 2020.

#### 2.8.2 Metastatic Potential Assay

The metastatic potential of the extracts was identified by using the XTT assay, developed by the Molecular Targets Program (MTP), Assay Development and Screening Section, National Cancer Institute (NCI), *via* comparing the effects of the extracts on high (MG63.3) and low (MG63) metastatic potential osteosarcoma cell lines. The cells were plated in tissue culture plates and allowed to attach overnight followed by a 2-day treatment with the extracts. Relative cell numbers are assessed using the XTT assay. The results were evaluated to determine which extract has activity ≤50% for MG63.3 and ≥50% for MG63, and the difference between the two values must be ≥ 50% (Eroglu Ozkan et al., 2020).

### 2.9 Statistical Analysis

All measurements were repeated three times. The results were evaluated using a *t*-test with Microsoft Excel and expressed as mean ± standard deviation. Differences were considered significant at *p* < 0.05.

## 3 Results and Discussion

### 3.1 LC-MS/MS Analysis of the Extracts

#### 3.1.1 LC-MS/MS Method Validation

The LC-MS/MS method validation was carried out by a previous study (Yilmaz et al., 2018). The details were given in the following sections.

##### 3.1.1.1 Linearity

The linearity was assayed using external standard calibration curve with six concentration levels for each analyte, and each concentration level was assayed in triplicate. The developed method showed to be linear for all compounds, between the ranges of tested concentrations during the validation of the method with *R* ^2^ ≥ 0.989. The equations for the calibration curves and the determination coefficients (*R* ^2^) are shown in Supplementary Table S2.

##### 3.1.1.2 Accuracy (Recovery) and Precision (Repeatability)

For intra-day variability assessment, spiked samples were measured for six replicates within a single day, whereas spiked samples were examined in triplicate per day for three consecutive days to conduct inter-day assay. As a result of the studies conducted on the same day and on different days, recovery and % RSD values were calculated to determine the accuracy and precision (Supplementary Table S2).

The recovery was calculated with the following equation:

Recovery (%) = (Amount Found-OriginalAmount)/Amount Spiked × 100%

##### 3.1.1.3 Limits of Detection and Quantification

To determine the LOD and LOQ values for the phytochemicals used in the LC-MS/MS method, analyte mixture was spiked at 10 identical samples prepared from a selected extract at the lowest concentration signaled by the standards and injected to the instrument.

LOD and LOQ values were calculated according to the following equations (Supplementary Table S2):

$$\begin{array}{l} \mathrm{LOD}\;=\;\text{Mean +}\;3\;\times \;\text{Standard Deviation }\\ \mathrm{LOQ}\;=\;\text{Mean +}\;\mathrm{10}\;\times \;\mathrm{Standard Deviation} \end{array}$$

##### 3.1.1.4 Relative Standard Uncertainty (U95)

Standard uncertainties of the analytes were determined by the accuracy (recovery) and precision (repeatability) studies according to EURACHEM Guide (2012).

#### 3.1.2 LC-MS/MS Analysis Results of the Extracts

As mentioned, the studied *T. praecox* subsp*. grossheimii* (Ronniger) Jalas, *T. pubescens* Boiss. et Kotschy ex Celak, and *T. cariensis* Hub-Mor. & Jalas samples were collected from Turkey. Five ethanol extracts were prepared from the collected samples (aerial parts and roots extracts from *T. praecox* subsp*. grossheimii* (Ronniger) Jalas, and *T. pubescens* Boiss. et Kotschy ex Celak, and aerial parts extract from *T. cariensis* Hub-Mor. & Jalas) and the extract yield of the samples were presented in Table 1. Aiming to identify their chemical compositions, An LC-MS/MS analysis was carried out, and the obtained results are present in Table 2.

**TABLE 2:** LC-MS/MS results of the studied *Thymus* extracts.

No	Analytes	Quantification (μg analyte/g extract)				
		Tc	TprA	TprR	TpuA	TpuR
1	Coumarin	N.D.	N.D.	N.D.	N.D.	N.D.
2	Hesperidin	N.D.	117.42 ± 3.08	N.D.	233.21 ± 6.11	42.82 ± 1.12
3	p-coumaric acid	98.55 ± 5.09	36.94 ± 1.91	28.00 ± 1.44	56.63 ± 2.92	56.93 ± 2.94
4	o-coumaric acid	N.D.	N.D.	N.D.	N.D.	N.D.
5	Gallic acid	N.D.	N.D.	N.D.	N.D.	N.D.
6	Caffeic acid	322.7 ± 11.42	250.74 ± 8.88	5.87 ± 0.21	632.06 ± 22.37	504.09 ± 17.84
7	Vanillic acid	N.D.	N.D.	N.D.	N.D.	N.D.
8	Salicylic acid	18.14 ± 0.60	37.6 ± 1.24	21.1 ± 0.69	83.04 ± 2.73	23.56 ± 0.78
9	Quinic acid	6548.64 ± 53.70	8,374.43 ± 68.67	1077.98 ± 8.84	7563.64 ± 62.02	2103.87 ± 17.25
10	4-OH-Benzoic acid	N.D.	N.D.	N.D.	N.D.	N.D.
11	tr-Ferulic acid	N.D.	N.D.	85.09 ± 4.20	N.D.	82.07 ± 4.05
12	Chlorogenic acid	N.D.	N.D.	N.D.	70.34 ± 0.49	N.D.
13	Rosmarinic acid	2501.3 ± 178.34	2166.60 ± 154.48	N.D.	2499.32 ± 178.20	1026.79 ± 73.21
14	Protocatechuic acid	1463.02 ± 60.13	96.68 ± 3.97	N.D.	231.39 ± 9.51	286.03 ± 11.76
15	Cinnamic acid	N.D.	N.D.	N.D.	N.D.	N.D.
16	Sinapinic acid	N.D.	N.D.	N.D.	N.D.	N.D.
17	Fumaric acid	1715.45 ± 21.27	552.98 ± 6.86	641.36 ± 7.95	637.82 ± 7.91	461.82 ± 5.73
18	Vanillin	28.05 ± 0.79	18.74 ± 0.52	163.68 ± 4.58	58.77 ± 1.65	162.24 ± 4.54
19	Pyrocatechol	N.D.	N.D.	N.D.	N.D.	N.D.
20	Malic acid	855.65 ± 9.67	624.59 ± 7.06	616.98 ± 6.97	1141.92 ± 12.90	510.17 ± 5.76
21	Syringic acid	N.D.	N.D.	N.D.	N.D.	N.D.
22	Hesperetin	2.37 ± 0.13	3.80 ± 0.21	8.48 ± 0.48	16.48 ± 0.93	13.19 ± 0.74
23	Naringenin	54.07 ± 2.82	163.62 ± 8.52	26.92 ± 1.40	2226.14 ± 115.98	119.06 ± 6.20
24	Rutin	35.25 ± 0.56	9.24 ± 0.15	0.59 ± 0.01	51.76 ± 0.82	9.29 ± 0.15
25	Quercetin	N.D.	N.D.	N.D.	N.D.	N.D.
26	Quercitrin	N.D.	N.D.	N.D.	256.98 ± 5.16	N.D.
27	Apigenin	161.5 ± 10.50	321.49 ± 20.90	94.81 ± 6.16	593.43 ± 38.57	96.09 ± 6.25
28	Chrysin	N.D.	N.D.	N.D.	2.72 ± 0.05	N.D.
29	Liquiritigenin	N.D.	N.D.	N.D.	N.D.	N.D.
30	Isoquercitrin	N.D.	22.81 ± 0.30	N.D.	205.75 ± 2.74	N.D.
31	Cosmosiin	N.D.	N.D.	N.D.	N.D.	N.D.
32	Rhoifolin	66.22 ± 6.23	N.D.	N.D.	542.43 ± 51.04	N.D.
33	Nicotiflorin	4.16 ± 0.11	244.43 ± 6.75	90.84 ± 2.51	1241.4 ± 34.26	244.51 ± 6.75
34	Fisetin	N.D.	N.D.	N.D.	N.D.	N.D.
35	Luteolin	N.D.	N.D.	N.D.	N.D.	N.D.
36	Myricetin	N.D.	N.D.	N.D.	N.D.	N.D.
37	Kaempferol	N.D.	N.D.	N.D.	67.32 ± 1.41	N.D.

Tc, *Thymus cariensis*; TprA, *Thymus praceox* subsp. *grossheimi* aerial part; TprR, *Thymus praceox* subsp. *grossheimi* root part; TpuA, *Thymus pubescens* aerial part; TpuR, *Thymus pubescens* root part; N.D, Not detected.

Twelve biologically important secondary metabolites were determined in all studied extracts with different concentrations: *p-*coumaric acid (28 ± 1.44–98.55 ± 5.09 μg/g extract), caffeic acid (5.87 ± 0.21–632.06 ± 22.37 μg/g extract), salicylic acid (18.14 ± 0.60–83.04 ± 2.73 μg/g extract), quinic acid (1077.98 ± 8.84–8,374.43 ± 68.67 μg/g extract), fumaric acid (461.82 ± 5.73–1715.45 ± 21.27 μg/g extract), vanillin (18.74 ± 0.52–163.68 ± 4.58 μg/g extract), malic acid (510.17 ± 5.76–1141.92 ± 12.90 μg/g extract), hesperetin (2.37 ± 0.13–16.48 ± 0.93 μg/g extract), naringenin (26.92 ± 1.40–2226.14 ± 115.98 μg/g extract), rutin (0.59 ± 0.01–51.76 ± 0.82 μg/g extract), apigenin (94.81 ± 6.16–593.43 ± 38.57 μg/g extract), and nicotiflorin (4.16 ± 0.11–1241.4 ± 34.26 μg/g extract).

In aerial part ethanol extract of *T. cariensis* Hub-Mor. & Jalas (TcA), 15 different compounds were detected. The extract was found to be the richest in terms of *p*-coumaric acid (98.55 ± 5.09 μg/g extract), rosmarinic acid (2501.3 ± 178.34 μg/g extract), protocatechuic acid (1463.02 ± 60.13 μg/g extract), and fumaric acid (1715.45 ± 21.27 μg/g extract). Moreover, the amounts of quinic acid (6548.64 ± 53.70 μg/g extract), malic acid (855.65 ± 9.67 μg/g extract), and caffeic acid (322.7 ± 11.42 μg/g extract) were found to be quite abundant in the extract. Salicylic acid (18.14 ± 0.60 μg/g extract), vanillin (28.05 ± 0.79 μg/g extract), hesperetin (2.37 ± 0.13 μg/g extract), naringenin (54.07 ± 2.82 μg/g extract), rutin (35.25 ± 056 μg/g extract), rhoifolin (66.22 ± 6.23 μg/g extract), and nicotiflorin (4.16 ± 0.11 μg/g extract) were the further constituents of TcA. In a previous report about the determination of phenolic compounds of the acetone and methanol extracts obtained from *T. cariensis* Hub-Mor. & Jalas by HPLC-DAD, luteolin (68.37 ± 3.62 mg/g), rosmarinic acid (26.51 ± 1.87–30.13 ± 2.63 mg/g), and fumaric acid (6.31 ± 0.42 mg/g) were the major compounds (Kucukaydin et al., 2021). Another study was conducted to investigate the main components of *T. cariensis* Hub-Mor. & Jalas hexane extract by GC-FID, and accordingly, linolenic acid (22.65%), linoleic acid (14.37%), behenic acid (12.54%), and palmitic acid (15.08%) were detected in the hexane extract, respectively (Kucukaydin et al., 2020).

*T. praecox* subsp*. grossheimii* (Ronniger) Jalas aerial parts extract (TprA) was shown to contain 16 different phytochemicals. The extract was the richest in terms of quinic acid with 8,374.43 ± 68.67 μg/g extract. The amounts of rosmarinic acid (2166.60 ± 154.48 μg/g extract), malic acid (624.59 ± 7.06 μg/g extract), fumaric acid (552.98 ± 6.86 μg/g extract), apigenin (321.49 ± 20.90 μg/g extract), caffeic acid (250.74 ± 8.88 μg/g extract), nicotiflorin (244.43 ± 6.75 μg/g extract), and naringenin (163.62 ± 8.52 μg/g extract) were also noteworthy in TprA. *T. praecox* subsp*. grossheimii* (Ronniger) Jalas roots extract (TprR) was revealed to consist of 13 compounds by this analysis. This was the most abundant extract in terms of tr-ferulic acid (85.09 ± 4.20 μg/g extract), and vanillin (163.68 ± 4.58 μg/g extract). *p*-coumaric acid (28 ± 1.44 μg/g extract), caffeic acid (5.87 ± 0.21 μg/g extract), salicylic acid (21.1 ± 0.69 μg/g extract), quinic acid (1077.98 ± 8.84 μg/g extract), fumaric acid (641.36 ± 7.95 μg/g extract), malic acid (616.98 ± 6.97 μg/g extract), naringenin (26.92 ± 1.40 μg/g extract), apigenin (94.81 ± 6.16 μg/g extract), and nicotiflorin (90.84 ± 2.51 μg/g extract) were also screened at meaningful amounts in the extract. Since *T. praecox* has several subspecies and varieties botanically, investigating their chemical compositions comparatively has been the goal of several previous studies, which are worth mentioning here in this context. For instance, a previous HPLC analysis of *T. praecox* subsp. *grossheimii* (Ronniger) Jalas *s*uggested that gallic acid, protocatechuic aldehyde, chlorogenic acid, caffeic acid, vanillin, ferulic acid, and rosmarinic acid were screened in the methanol extract of the plant (Burnaz et al., 2017). In another study, ursolic acid, oleanolic acid rosmarinic acid and its derivatives, luteolin 5-O-β-D-glucopyranoside, and thymoquinol 2,5-O-β-diglucopyranoside were isolated from the aerial parts of *T. praecox* subsp*. grossheimii* var. *grossheimii,* which is another variety of the plant (Sevindik et al., 2015). Furthermore, rosmarinic acid, caffeic acid, luteolin-7-rutinoside, luteolin-7-glucoside, and apigenin-7-glucoside were found to be present in ethanol extract of another *T. praecox* subspecies, namely *Thymus praecox* ssp. *arcticus* (E. Durand), Jalas by HPLC–UV (Raudone et al., 2017). Another HPLC-UV analysis was carried out this time on *T. praecox* subsp*. caucasicus* var*. caucasicus*, indicating that quercetin, luteolin, and caffeic acid were the major constituents in the studied extracts along with different phenolic compounds such as p-coumaric acid, protocatechuic acid, and catechin (Turumtay et al., 2014). The screening of *T. praecox* subsp. *skorpilii* var. *skorpilii* (Velen.) Jalas methanol extract by HPLC-DAD and LC-MS/MS techniques showed the presence of chlorogenic acid, luteolin-7-O-glucoside, 3-O-feruloylquinic acid, quercetin-3-O-hexoside, and apigenin-7-O-glucuronide (Taskin et al., 2019).

The aerial parts extract of *T. pubescens* Boiss. et Kotschy ex Celak (TpuA) is undoubtedly the richest extract among the five studied, consisting of 21 different biologically active phytochemicals. Incidentally, quercitrin (256.98 ± 5.16 μg/g extract), chlorogenic acid (70.34 ± 0.49 μg/g extract), kaempferol (67.32 ± 1.41 μg/g extract), and chrysin (2.72 ± 0.05 μg/g extract) were the compounds that are only present in this extract. On top of that, being the richest in terms of naringenin (2226.14 ± 115.98 μg/g extract), caffeic acid (632.06 ± 22.37 μg/g extract), apigenin (593.43 ± 38.57 μg/g extract), hesperidin (233.21 ± 6.11 μg/g extract), malic acid (1141.92 ± 12.90 μg/g extract), hesperetin (16.48 ± 0.93 μg/g extract), rutin (51.76 ± 0.82 μg/g extract) isoquercitrin (205.75 ± 2.74 μg/g extract) rhoifolin (542.43 ± 51.04 μg/g extract), and nicotiflorin (1241.4 ± 34.26 μg/g extract), TpuA can be considered as a valuable source in the search for plant-derived natural compounds. In the root extract of the plant (TpuR), 16 secondary metabolites were shown to be present. The extract was to contain a significant amount of important phenolic acids, such as quinic acid (2103.87 ± 17.25 μg/g extract), rosmarinic acid 1026.79 ± 73.21 μg/g extract), malic acid (510.17 ± 5.76 μg/g extract), caffeic acid (504.09 ± 17.84 μg/g extract), and fumaric acid (461.82 ± 5.73 μg/g extract). Although the data about the chemical composition of *T. pubescens* Boiss. et Kotschy ex Celak extracts are rather scarce and the research is mainly focused on its volatile compounds, according to a previous study, rosmarinic acid was the main constituent of *T. pubescens* Boiss. et Kotschy ex Celak, and gallic acid, caffeic acid, epicatechin, luteolin*, p*-coumaric acid, ferulic acid, cinnamic acid, apigenin, naringenin, and salvianolic acid were also detected in the plant extract (Sarfaraz et al., 2021).

As known, the pharmacological activities of the plants are attributed to their naturally occurring phytochemicals. Polyphenols, in particular, have shown to possess significant pharmacological properties that are effective for modulating human metabolism in a manner favorable for the treatment of several ailments, as well as prevention or reduction in the risk of degenerative diseases. Within this framework, reported by the current study for the first time, the studied *Thymus* species showed the important presence of various phenolic compounds that could be beneficial for human health.

### 3.2 Total Phenolic and Flavonoid Contents of the Extracts

Total phenolic contents of the extracts were calculated from pyrocatechol calibration curve (*y* = 0.0408*x* + 0.0549, *R* ^2^ = 0.9924) and expressed as µgs of pyrocatechol equivalents (PEs), and total flavonoid contents of the extracts were calculated from quercetin calibration curve (*y* = 0.0325*x* + 0.0601, *R* ^2^ = 0.9984), and expressed as µgs of quercetin equivalents (QEs). The results are shown in Table 3.

**TABLE 3:** Total phenolic, flavonoid contents, antioxidant activities, and enzyme inhibition activities of the studied *Thymus* extracts^*^.

Samples	Phenolic content (*μ*g PEs/mg extract)^a^	Flavonoid content (*μ*g QEs/mg extract)^b^	DPPH free radical IC_50_ (µg/ml)	ABTS cation radical IC_50_ (µg/ml)	CUPRAC A_0.5_ (µg/ml)	AChE (inhibition %, 200 μg/ml)	BChE (inhibition %, 200 μg/ml)	Tyrosinase (inhibition %, 200 μg/ml)	Urease (inhibition %, 200 μg/ml)
Tc	61.12 ± 2.59	17.18 ± 1.04	34.97 ± 1.00	9.98 ± 0.04	5.80 ± 0.02	NA	35.61 ± 1.20	NA	NA
TprA	39.34 ± 1.73	21.20 ± 0.62	91.42 ± 0.71	50.32 ± 0.60	22.96 ± 0.38	NA	10.26 ± 0.70	NA	NA
TprR	11.15 ± 0.17	2.53 ± 0.04	248.61 ± 2.44	69.28 ± 1.71	28.03 ± 0.13	NA	7.07 ± 0.08	NA	NA
TpuA	45.47 ± 0.57	40.28 ± 0.92	51.44 ± 2.08	10.54 ± 0.09	9.73 ± 0.05	NA	33.49 ± 1.39	NA	NA
TpuR	52.82 ± 1.47	9.35 ± 0.65	41.80 ± 0.31	13.48 ± 0.16	10.81 ± 0.07	NA	15.78 ± 0.82	NA	NA
BHA^c^	—	—	7.88 ± 0.20	2.74 ± 0.03	0.63 ± 0.02	—	—	—	—
α-TOC^c^	—	—	16.30 ± 0.79	10.20 ± 0.05	13.38 ± 0.07	—	—	—	—
BHT^c^	—	—	58.86 ± 0.50	3.16 ± 0.06	2.02 ± 0.01	—	—	—	—
Galanthamine^c^	—	—	—	—	—	83.31 ± 0.09	86.38 ± 0.10	—	—
Kojik acid^c^	—	—	—	—	—	—	—	95.26 ± 0.23	—
Tiyourea^c^	—	—	—	—	—	—	—	—	88.61 ± 1.16

Tc, *Thymus cariensis*; TprA, *Thymus praceox* subsp. *grossheimi* aerial part; TprR, *Thymus praceox* subsp. *grossheimi* root part; TpuA, Thym*us pubescens* aerial part; TpuR, *Thymus pubescens* root part; NA, Not Active.

* Values expressed are means ± standard deviation of three parallel measurements (*p* < 0.05).

a PEs, pyrocatechol equivalents (*y* = 0.0408*x* + 0.0549. *R* ^2^ = 0.9924).

b QEs, quercetin equivalents (*y* = 0.0325*x* + 0.0601. *R* ^2^ = 0.9984).

c Standart compounds.

Regarding the phenolic content, TcA was found as the richest extract with 61.12 ± 2.59 μg PEs/mg extract. *T. pubescens* Boiss. et Kotschy ex Celak extracts were reported to contain a higher amount of phenolic compounds compared to the *T. praecox* subsp. *grossheimii* (Ronniger) Jalas extracts. TpuR was the richest extract among them with 52.82 ± 1.48 μg PEs/mg extract. TpuA (45.47 ± 0.57 μg PEs/mg extract), TprA (39.34 ± 1.73 μg PEs/mg extract), and TprR (11.15 ± 0.17 μg PEs/mg extract) were also found to contain good amount of polyphenols. In terms of flavonoid content, TpuA was the richest extract with 40.28 ± 0.92 μg QEs/mg extract. Total flavonoid contents of other extracts were determined as TprA: 21.20 ± 0.62 μg QEs/mg extract, TcA: 17.18 ± 1.04 μg QEs/mg extract, TpuR: 9.35 ± 0.65 μg QEs/mg extract, and TprR: 2.53 ± 0.04 μg QEs/mg extract, respectively.

Taking a look at the previous studies conducted in this field, the total phenolic and flavonoid contents of the extracts of *T. cariensis* Hub-Mor. & Jalas and *T. cilicicus* Boiss. & Balansa were calculated. The phenolic contents of the extracts were reported to range from 42.74 ± 0.12 to 113.61 ± 0.15 μg PEs/mg, and the flavonoid contents of the extracts were reported to range from 29.57 ± 0.01 to 175.47 ± 0.21 μg QEs/mg (Kucukaydin et al., 2021). *T. pubescens* Boiss. et Kotschy ex Celak were found to contain 295.57 ± 1.91 μg rutin/mg extract of phenolic compounds, and 50.39 ± 0.75 μg rutin/mg extract of flavonoids (Nickavar and Esbati, 2012). In another study, total phenolic and flavonoid contents of the extracts were obtained separately from the leaves, flowers, and stems of *T. praecox* subsp. *caucasicus* var. *caucasicus* (Willd. ex Ronniger) Jalas were calculated. The highest phenolic content was determined in the hydrolyzed-flower extract with 27.634 ± 1.677 mg GAE g^−1^, and the highest flavonoid content was determined in the hydrolyzed-leaf extract with 23.639 ± 1.06 mg QE g^−1^. As expected, the standard mix extracts with the total plant were found to contain the highest amount of phenolics (1341.743 ± 18.710 mg GAE g^−1^), and flavonoids (380.519 ± 5.132 mg QE g^−1^) (Turumtay et al., 2014). Other *Thymus* L. species were also subjected to numerous studies and their total phenolic and flavonoid contents were calculated. For instance, *T. convolutus* Klokov, *T. fallax* Fisch. & C. A. Mey*., T. haussknechtii* Velen*, T. kotschyanus* var. *kotschyanus* Boiss. & Hohen, and *T. sipyleus* subsp. *sipyleus* var. *sipyleus* Boiss from Turkey were investigated in this context, phenolic contents of the extracts were found to range from 35.25 ± 0.75 to 96.58 ± 0.86 μg PEs/mg extract, and flavonoid contents of the extracts were shown to range from 1.71 ± 0.06 to 45.30 ± 0.84 μg QEs/mg extract (Boga et al., 2021a). Although the identification of phenolic compounds as the constituents of different plant extracts could only be possible by specific analytical techniques including LC-MS/MS or HPLC, using low-cost photometric assays such as the methods used in this study, it is possible to determine the sub-groups sharing basic chemical structures (Csepregi et al., 2013). To evaluate the antioxidant potential of the extracts, calculating the total phenolic and flavonoid contents is a crucial step since the antioxidant activity is usually attributed to the phenolics present in the extracts, and is considered a hallmark for important biological activities (Ersoy et al., 2020; Eroglu Ozkan et al., 2021).

### 3.3 Antioxidant Activity of the Extracts

Three different methods were applied to investigate the *in vitro* antioxidant activity of the studied *Thymus* extracts. Using various methods and evaluating all the gathered data altogether are crucial steps to considering every oxidation aspect (Boga et al., 2021b) The results are presented in Table 3.

TcA demonstrated the strongest antioxidant activity in all three assays. According to the DPPH radical scavenging activity assay results, the IC_50_ value of TcA was 34.97 ± 1.00 μg/ml, which is higher than the other four extracts and also one of the standard compounds, namely BHT (58.86 ± 0.50 μg/ml). ABTS cation radical assay was the second procedure, and TcA (9.98 ± 0.04 μg/ml) showed stronger antioxidant activity than the standard compound α-TOC (10.20 ± 0.05 μg/ml), and the other three extracts once again. Similar results were obtained from the CUPRAC assay, A_0.5_ values were calculated here and TcA (5.80 ± 0.02 μg/ml) was found to be more active than the standard α-TOC (13.38 ± 0.07 μg/ml) and the other extracts. On the other hand, all *T. pubescens* Boiss. et Kotschy ex Celak extracts appeared to possess better antioxidant capacities than *T. praceox* subsp. *grossheimii* (Ronniger) Jalas extracts according to the results of all three assays.

Understanding the antioxidant properties of thyme is of utmost importance because the antioxidant molecules play a protective role in the pathogenesis of major disease burdens such as cancer, Alzheimer’s, or diabetes mellitus. The balance between the oxidative and reducing species must be maintained in healthy humans, where oxidative stress leads to the overproduction of reactive species, therefore causing the imbalance and emerging diseases. Even supposing antioxidant supplements cannot be served as medications, it must be noted that they indirectly help to combat these diseases and consequently be beneficial for the healthspan and lifespan extension (Conti et al., 2016; Tan et al., 2018).

Total phenolic content has been reported to be a good predictor of the antioxidant activity of plant extracts, which is positively correlated with antioxidant activity (Li et al., 2018). The current study also showed parallelism with this finding since the extract of *T. cariensis* Hub-Mor. & Jalas contained the highest total phenolic content and demonstrated the strongest antioxidant activity among all studied extracts. The antioxidant potentials of various *Thymus* species have been investigated in countless studies. Although the research has mainly been focused on the essential oil of thyme, extracts, and isolated compounds such as thymol have also been investigated for their antioxidant capacities (Dauqan et al., 2017; Nagoor Meeran et al., 2017; Salehi et al., 2018; Lorenzo et al., 2019). Regarding all data acquired from these studies, thyme in general is much more important than a basic nutrient or a taste enhancer only. With its rich phenolic content and strong antioxidant properties, thyme has been revealed as a food preservative and also a functional food that provides outstanding benefits to human health (Boga et al., 2021a). Incidentally, it has been confirmed by a recent study that the infusion and decoction of thyme could be achieved by internal as well as external use for the treatment of severe diseases caused by the reactive oxygen, nitrogen, and sulfur species production and oxidative stress with its antioxidant activity and unique phenolic characterization (Martins et al., 2015).

It must be noted that there are divergent opinions about the possibility of predicting the antioxidant activity of the plant extracts by employing *in vitro* antioxidant screening methods, which are mainly colorimetric methods that have widespread applications (Boga et al., 2021a). Although recent studies have shown that colorimetric methods are still considered as very important tools in terms of antioxidant activity investigation, further studies are required to prove more solid evidence in this regard.

### 3.4 Enzyme Inhibition Activity of the Extracts

The enzyme inhibitory potentials of TcA, TprA, TprR, TpuA, and TpuR were assessed by antiacetylcholinesterase, antibutyrylcholinesterase, antityrosinase, and antiurease activity assays at 200 μg/ml concentration. The results are given in Table 3.

The anticholinesterase activity of the extracts was investigated using Ellman’s colorimetric method. Accordingly, the studied *Thymus* extracts did not demonstrate any inhibitory activity on the acetylcholinesterase enzyme. Taking a glance at previous studies in this regard, *T. cariensis* Hub-Mor. & Jalas extracts prepared with different solvents were found to show moderate to low anticholinesterase activity. For instance, the IC_50_ value of the methanol extract was 68.65 ± 0.08 μg/ml, whereas the acetone extract inhibited AChE with 87.10 ± 0.25 μg/ml, and the hexane extract with 190.2 ± 0.18 μg/ml, respectively (Kucukaydin et al., 2021). There have also been several studies carried out to evaluate the AChE inhibitory potential of different *Thymus* species, and they were shown to exert antiacetylcholinesterase activity in a dose-dependent manner (Kindl et al., 2015; Taskin et al., 2019). However, after a more detailed literature survey, it has appeared that the ethanol extracts of Thymus species were found to demonstrate very low or no inhibitory effect on AChE. In Orhan et al.’s study, dichloromethane and ethyl acetate extracts of *T. praecox* subsp. *caucasicus* var. *caucasicus* was shown to be moderately active, whereas the ethanol extract was comparatively much less effective (Orhan et al., 2009). In Ertas et al.’s study, methanol extract of *T. nummularius* M. Bieb. demonstrated weak AChE inhibitory activity with 13.31 ± 0.21% at 200 μg/ml, however, the antiacetylcholinesterase potential of the essential oil of the plant was higher with 60.05 ± 0.37%, which was concluded by the discussion that this difference was attributed to a specific component, thymol, which was present in the essential oil but not in the extract of *T. nummularius M. Bieb.* (Ertas et al., 2015). Ceylan et al. (2016) also indicated that the presence of thymol and carvacrol is required for anti-AChE activity. Another example of this situation could be observed in Bendjabeur et al. (2018)’s study as well, where *T. algeriensis* Boiss. & Reut ethanol extract was completely inactive on AChE, but the essential oil was weakly active on the same enzyme with an IC_50_ value of 98.84 ± 1.81 μg/ml. Moreover, ethanol extracts of the aerial parts and roots of five different thyme species were also shown to have no AChE inhibitory potential (Boga et al., 2021a). This may explain the lack of activity.

Differently from AChE inhibition activity results, all extracts could inhibit BChE weakly or moderately (7.07 ± 0.08–35.61 ± 1.20%). The abovementioned studies except for Orhan et al. (2009)’s study, investigate the antibutyrylcholinesterase potential of different *Thymus* species as well. It can be seen the extracts were slightly more effective on BChE in comparison to their anti-AChE effects.

The studied five *Thymus* extracts were found to show no inhibitory activity on both tyrosinase and urease enzymes (Table 4). Same as the anticholinesterase activity, essential oils obtained from different *Thymus* species were shown to possess antityrosinase potential owing to a high level of thymol present in the oils (Ceylan et al., 2016). *T. argaeus* (Fisch. & C.A.Mey.) Boiss. & Balansa methanolic extract was found to exert anti-tyrosinase activity with 121.52 mg KAE/g sample, whereas the decoction of the plant exerted lesser activity with 55.29 mg KAE/g sample (Zengin et al., 2019). Galuteolin, a secondary metabolite that was found in the flower extract of *T. quinquecostatus* Celak. demonstrated significant anti-tyrosinase activity (Kim et al., 2021). In another study, again phytochemicals isolated from *T. vulgaris* L. such as 6′,3′,4′-trihydroxy-5,5′-diisopropyl-2,2′-dimethylbiphenyl, thymol, oleanolic acid, ursolic acid, cirsimaritin, and xanthomicrol were capable of inhibiting tyrosinase, which also proves only specific compounds are responsible for the tyrosinase inhibitory activity and this activity is only expected when they are present in the extracts (Stefanis et al., 2019). The same conclusion is also accurate for the anti-urease activity and reported by Boga et al. (2021a)’s study again, no tyrosinase urease activity was demonstrated by the investigated ten *Thymus* L*.* ethanol extracts.

**TABLE 4:** Cytotoxic activity results of studied *Thymus* extracts.

Exracts	2 Days-Renal (viability%)		2 Days-Colon (viability%)		META (viability %)	
	A498	UO31	COLO205	KM12	MG633	MG63
Tc	NA	NA	NA	NA	NA	NA
TprA	61.92	89.96	81.13	NA	96.04	84.94
TprR	81.95	88.37	68.43	75.71	NA	90.46
TpuA	65.31	96.87	93.46	96.4	99.74	96.99
TpuR	68.33	93.41	81.87	NA	NA	91.05

Tc, *Thymus cariensis*; TprA, *Thymus praceox* subsp. *grossheimi* aerial part; TprR, *Thymus praceox* subsp. *grossheimi* root part; TpuA, *Thymus pubescens* aerial part; TpuR, *Thymus pubescens root* part; NA, Not Active.

### 3.5 Cytotoxic Activity and Metastatic Potential of the Extracts

The cytotoxic potential of the extracts was evaluated on COLO205 and KM12 human colon, UO-31, and A498 human renal cancer cell lines, and the metastatic potential of the extracts was evaluated on MG633 high metastatic and MG63 low metastatic osteosarcoma cancer cell lines by cell viability assays. The results were summarized in Table 4. Evaluating the results, it can be seen that TprA exhibited moderate cytotoxic activity on A498 renal cell lines with 61.92% cell viability. Roots extract of the same plant, TprR also exerted moderate cytotoxic activity on COLO205 human colon cancer cell lines with 68.43% cell viability after 2 days. TpuA and TpuR extracts were also effective in terms of cytotoxicity on A498 human renal cancer cell lines with 65.31% and 68.33%, respectively. On KM12 colon cancer cell lines, only TprR extract demonstrated moderate-to-low cytotoxic activity with 75.71%. None of the extracts was shown to possess metastatic potential, also *T. cariensis* Hub-Mor. & Jalas extract was found to be ineffective on all the studied cell lines.

Despite the fact that enormous scientific endeavors have been made to have a better understanding of cancer, which is the second leading cause of death, in order to combat this disease, there is still an urgent need for further anti-cancer agents. Natural and plant-derived compounds with their anti-cancer properties have aroused the attention of researchers in this context. *Thymus* species with their well-known pharmacological effects and unique chemistry have also been investigated for their cytotoxic activity. Although the anti-cancer research has been mainly focused on different essential oils obtained from different *Thymus* species, recently the extracts of these plants have also been studied. *T. vulgaris L.*, the undoubtedly most investigated species, was shown to have a cytotoxic effect on H460 large cell lung carcinoma, THP-1 human leukemia, Caco-2 human colon adenocarcinoma, and HepG2 human hepatocellular carcinoma cell lines (Ayesh et al., 2014; Oliviero et al., 2016; Taghouti et al., 2020). *T. caramanicus* Jalas demonstrated cytotoxic activity on KB human oral epidermoid carcinoma cell lines, also the extract was shown to potentiate the effect of the anti-cancer drug, doxorubicin (Fekrazad et al., 2017). *T. kotschyanus* var. *kotschyanus* extract was found to decrease the cell viability of A498 renal cancer cell lines by 61.77% in another study (Boga et al., 2021a). *T. cariensis* Hub-Mor. & Jalas was also studied for its cytotoxic activity. Methanol and acetone extracts were shown to have anti-cancerous effects on MCF-7 breast cancer cell lines, whereas the hexane extract was found to be ineffective (Kucukaydin et al., 2021a). This is important because it proves the direct effect of the extracting solvent on the cytotoxic activity. Another noteworthy finding was in terms of cytotoxicity, thyme extracts could potentially be active on human cancer cell lines but not on healthy cells. This selectiveness is rather meaningful and requires further studies to understand the pathways mediating the anti-cancer effect (Patil et al., 2021).

### 3.6 Antimicrobial Activity of the Extracts

The extracts, TcA, TprA, TprR, TpuA, and TpuR were investigated for their antimicrobial activity against a standard panel of pathogenic bacteria and fungi. The results are given as MIC values, and they are shown in Table 5.

**TABLE 5:** Antimicrobial activity results of the studied *Thymus* extracts.

Microorganisms	MIC values (µg/ml)				
	Tc	TprA	TprR	TpuA	TpuR
*Pseudomonas aeruginosa* ATCC 27853	NA	NA	NA	NA	NA
*Escherichia coli* ATCC 25922	625	NA	NA	NA	NA
*Klebsiella pneumoniae* ATCC 4352	NA	NA	NA	NA	NA
*Proteus mirabilis* ATCC 14153	NA	NA	NA	NA	NA
*Staphylococcus aureus* ATCC 29213	NA	NA	NA	312.5	NA
*Staphylococcus epidermidis* ATCC 12228	1250	1250	1250	1250	625
*Enterococcus faecalis* ATCC 29212	625	1250	1250	1250	1250
*Candida albicans* ATCC 10231	NA	NA	NA	NA	NA
*Candida parapsilosis* ATCC 22019	312.5	NA	NA	312.5	312.5
*Candida tropicalis* ATCC 750	39.06	39.06	78.12	19.53	39.06

Tc, *Thymus cariensis*; TprA, *Thymus praceox* subsp. *grossheimi* aerial part; TprR, *Thymus praceox* subsp. *grossheimi* root part; TpuA, *Thymus pubescens* aerial part; TpuR, *Thymus pubescens* root part; NA, No Activity. Standards; Cefuroxime-Na 1.2 µg/ml for *S. aureus* ATCC 29213. Cefuroxime 9.8 µg/ml for *S. epidermidis* ATCC 12228. Amikacin 128 µg/ml for *E. faecalis* ATCC 29212. Ceftazidime 2.4 µg/ml for *P. aeruginosa* ATCC 27853. Cefuroxime-Na 4.9 µg/ml for E. coli ATCC 25922 and *K. pneumoniae* 4352. Cefuroxime-Na 2.4 µg/ml for *P. mirabilis* ATCC 14153. Clotrimazole 4.9 µg/ml for *C. albicans* ATCC 10231. Amphotericin B 0.5 µg/ml for *C. parapsilosis* ATCC 22019. Amphotericin B 1 µg/ml for *C. tropicalis* ATCC 750.

All studied extracts were found to be significantly effective on *C. tropicalis*, which is a well-known human pathogen associated with quite high mortality rates (Boga et al., 2021a). In terms of antifungal activity against *C. tropicalis*, TpuA was the most effective with 19.53 μg/ml, and TcA, TprA, TpuR were the second most effective with 39.06 μg/ml. TprR also exhibited antifungal activity against *C. tropicalis* with 78.12 μg/ml.

In previous studies, *T. mastichina* (L.) L. showed antimicrobial activity against several bacteria such as *E. coli*, *P. mirabilis*, *Salmonella* subsp*.*, *S. aureus*, MRSA, *L. monocytogenes EGD*, *B. cereus*, *Candida* spp*.*, and *Fusarium* spp. (Rodrigues et al., 2020). The nonpolar extracts of the aerial parts *T. eigii* were found to exert moderate antimicrobial activity, whereas polar extracts did not show any activity against a standard panel of pathogenic bacteria and fungi (Tepe et al., 2004). In another study, antimicrobial activity of *T. boissieri* Halácsy*, T. longicaulis* C. Presl*, T. leucospermus* Hartvig, and *T. ocheus* Heldr. & Sart. ex Boiss. extracts. The extracts exerted moderate antimicrobial activity, however, after encapsulating the extracts in liposomes, the antimicrobial effect was significantly increased. This may be a useful technique when stronger antimicrobial efficacy was aimed from *Thymus* extracts (Gotzi et al., 2006). Reviewing the literature about the antimicrobial activity of the essential oils and extracts obtained from *Thymus* species, it can be observed that essential oils demonstrate superior antimicrobial activity compared to the extracts. Speaking of which, a study about the evaluation of *T. vulgaris* L. essential oil and plant extracts for antimicrobial properties indicated that the extracts did not show any activity, whereas essential oil was found to possess antimicrobial properties (Gedikoglu et al., 2019). Essential oil and methanol extract of *T. hirtus* subsp. *algeriensis* (Boiss. and Reut.) Murb were also investigated for their antimicrobial activities and the results showed parallelism with the other findings and only the essential oil exhibited inhibitory activity on the growth of both Gram (+) and Gram (−) bacteria (Fatma et al., 2014). *T. daenensis* Celak is also among *Thymus* L. species with proven antimicrobial properties, whose essential oil was found to be more effective than several extracts (Zarshenas and Krenn, 2015). In a nutshell, it is a very well-known fact that essential oils obtained from *Thymus* L. species are not only better investigated, but also stronger antimicrobial agents than other *Thymus* extracts.

## 4 Conclusion

With the help of newly developed techniques in the field of related sciences, important pharmacological properties of different *Thymus* L. species including have been proved and more light was shed on their unique chemical characterizations. The current study provides data about chemical profiling and biological activities of three species namely *T. cariensis* Hub-Mor. & Jalas, *T. praceox* subsp. *grossheimii* (Ronniger) Jalas, and *T. pubescens* Boiss. et Kotschy ex Celak from Turkey. All studied extracts were shown to contain pharmacologically valuable phytochemicals such as hesperetin (2.37 ± 0.13–16.48 ± 0.93 μg/g extract), naringenin (26.92 ± 1.40–2226.14 ± 115.98 μg/g extract), rutin (0.59 ± 0.01–51.76 ± 0.82 μg/g extract), apigenin (94.81 ± 6.16–593.43 ± 38.57 μg/g extract), and demonstrate important biological activities including antioxidant, antityrosinase and antimicrobial effects, meaning these species could be beneficial for human health in various aspects. Since screening chemical constituents with the assessment of the biological activities are the crucial steps to having a better understanding towards new possible therapeutic agents derived from *Thymus* L. species, this assessment may contribute to the related sciences including pharmacognosy. Having said that, further studies including clinical trials are undoubtedly required to shed more light on the therapeutic properties of these species.

## Data Availability

The original contributions presented in the study are included in the article/Supplementary Material, further inquiries can be directed to the corresponding authors.

## Glossary

LC-MS/MS: Liquid Chromatography with Tandem Mass Spectrometry
QEs: Quercetin equivalents
PEs: Pyrocatechol equivalents
CUPRAC: Cupric Reducing Antioxidant Capacity
IC50: Concentration of drug required for 50% inhibition
DPPH: 2,2-diphenyl-1-picrylhydrazyl
ABTS: 2.2'-azino-bis(3-ethylbenzothiazoline-6-sulfonic acid
A0.5: The absorbance value at 0.5 concentration
MIC: Minimum Inhibitory Concentration
ESCOP: European Scientific Cooperative on Phytotherapy
WHO: World Health Organization
ISTE: The Herbarium of the Faculty of Pharmacy of Istanbul University
UHPLC: Ultra High Performance Liquid Chromatography
ESI: Electrospray Ionization
CLSI: Clinical and Laboratory Standards Institute
MTP: Molecular Targets Program
NCI: National Cancer Institute
MG63: Low Metastatic Potential Osteosarcoma Cell Lines
MG63.3: High Metastatic Potential Osteosarcoma Cell Lines
UO-31: Human Renal Cancer Cell Lines
A498: Human Renal Cancer Cell Lines
COLO205: Human Colon Cancer Cell Lines
KM12: Human Colon Cancer Cell Lines
BHT: Butylhydroxytoluene
BHA: Butylhydroxyanisole
ATCC: American Type Culture Collection
HPLC-DAD: High-Performance Liquid Chromatography with Diode-Array Detection
GAE: Gallic acid equivalents
α-TOC: Alpha-Tocopherol
TcA: *Thymus cariensis* Hub-Mor. & Jalas aerial parts extract
TprA: *Thymus praceox* subsp. *grossheimii* (Ronniger) Jalas aerial parts extract
TprR: *Thymus praceox* subsp. *grossheimii* (Ronniger) Jalas roots extract
TpuA: *Thymus pubescens* Boiss. Et Kotschy ex Celak aerial parts extract
TpuR: *Thymus pubescens* Boiss. et Kotschy ex Celak roots extract
AChE: Acetylcholinesterase
BChE: Butyrylcholinesterase
KAE: Kojic acid equivalents
MRSA: Methicillin-resistant *Staphylococcus aureus*
