# Supplementary material for: The Therapeutic Potential of Ethnomedicinally Important Anatolian Thyme Species: A Phytochemical and Biological Assessment
Source: Front Pharmacol. 2022 Jun 9;13:923063. doi: 10.3389/fphar.2022.923063 (PMC9218417; doi:10.3389/fphar.2022.923063)
Supplement: Supplementary file 1 [file DataSheet1.docx]

| **No** | **Analytes** | **RT^a^** | **Parent ion (m/z)^b^** | **Daughter Ions** | **Ion. Mode** | **Equation** | **R^2c^** | **RSD%^d^** | | **Linearity Range (µg/L)** | **LOD/LOQ (µg/L)^e^** | **Recovery (%)** | | **U^f^** |
| --- | --- | --- | --- | --- | --- | --- | --- | --- | --- | --- | --- | --- | --- | --- |
|  |  |  |  |  |  |  |  | **Intraday** | **Interday** |  |  | **Intraday** | **Interday** |  |
| 1 | Coumarin | 17,40 | 147,05 | 91,0-103,2 | Poz | y=33.6376×-897.142 | 0.994 | 0.01306 | 0.01239 | 1000-20000 | 208.49/228.38 | 0.99947 | 1.00081 | 0.0237 |
| 2 | Hesperidin | 12,67 | 610,90 | 303,1-465,1 | Poz | y=1340.27×-43769.1 | 0.998 | 0.00945 | 0.01126 | 25-1000 | 3.41/4.17 | 1.01733 | 1.01263 | 0.0262 |
| 3 | p-Couumaric acid | 11,53 | 162,95 | 119,25-93,25 | Neg | y=3199.2×+130019 | 0.992 | 0.01820 | 0.01727 | 25-1000 | 7.31/9.07 | 1.00617 | 1.01224 | 0.0516 |
| 4 | o- Couumaric acid | 15,45 | 162,95 | 119,35-93,25 | Neg | y=1219.34×-10915.7 | 0.999 | 0.02730 | 0.02566 | 25-1000 | 24.36/31.10 | 0.98344 | 0.99061 | 0.0513 |
| 5 | Gallic acid | 3,00 | 168,85 | 125,2-79,2 | Neg | y=226.763×+38152.3 | 0.998 | 0.01601 | 0.01443 | 250-10000 | 95.45/106.85 | 1.00004 | 1.00454 | 0.0282 |
| 6 | Caffeic acid | 8,80 | 178,95 | 135,2-134,3 | Neg | y=3963.32×+178156 | 0.998 | 0.01454 | 0.01469 | 25-1000 | 18.44/22.43 | 1.00917 | 0.98826 | 0.0354 |
| 7 | Vanilic acid | 8,57 | 166,90 | 152,25-108,25 | Neg | y=35.8398×-12097.9 | 0.999 | 0.00528 | 0.00619 | 1000-20000 | 122.21/139.70 | 1.00093 | 1.04095 | 0.0508 |
| 8 | Salicylic acid | 11,16 | 136,95 | 93,3-65,3 | Neg | y=5286.26×+309192 | 0.989 | 0.01016 | 0.01242 | 25-1000 | 5.00/6.53 | 1.00989 | 0.99013 | 0.0329 |
| 9 | Quinic acid | 1,13 | 190,95 | 85,3-93,3 | Neg | y=41.0559×+10671.6 | 0.996 | 0.00259 | 0.00274 | 250-10000 | 75.75/79.41 | 1.00288 | 0.98778 | 0.0082 |
| 10 | 4-OH-benzoic acid | 7,39 | 136,95 | 93,3-65,3 | Neg | y=409.028×+112079 | 0.998 | 0.01284 | 0.01538 | 250-10000 | 33.18/37.95 | 0.99662 | 1.00058 | 0.0289 |
| 11 | Ferrulic acid | 12,62 | 192,95 | 178,3 | Neg | y=80.453×-31782.5 | 0.997 | 0.00708 | 0.00619 | 250-10000 | 36.61/42.00 | 0.99987 | 1.00289 | 0.0494 |
| 12 | Chlorogenic acid | 7,13 | 353,15 | 191,2 | Neg | y=781.364×-18697.9 | 0.998 | 0.00058 | 0.00076 | 25-1000 | 6.19/8.11 | 1.00806 | 0.99965 | 0.0069 |
| 13 | Rosmarinic acid | 14,54 | 359 | 161,2-197,2 | Neg | y=909.672×-201692 | 0.994 | 0.02014 | 0.01751 | 100-5000 | 6.60/8.84 | 0.99206 | 1.03431 | 0.0713 |
| 14 | Protocatechuic acid | 4,93 | 152,95 | 108,3 | Neg | y=297.752×+30590.7 | 0.995 | 0.01236 | 0.01296 | 100-5000 | 28.24/31.39 | 0.99404 | 1.01070 | 0.0411 |
| 15 | Cinnamic acid | 25,61 | 147,00 | 103,15-77,3 | Neg | y=9.06322×-12403.2 | 0.996 | 0.00648 | 0.00816 | 5000-20000 | 839.84/915.08 | 1.00051 | 0.99927 | 0.0143 |
| 16 | Sinapinic acid | 12,66 | 222,95 | 208,3-149,2 | Neg | y=141.955×-73293.9 | 0.992 | 0.01446 | 0.01517 | 250-10000 | 77.59/85.51 | 1.00164 | 0.99962 | 0.0281 |
| 17 | Fumaric acid | 1,48 | 115,00 | 71,4 | Neg | y=64.9967×-11592.4 | 0.997 | 0.00536 | 0.00460 | 100-5000 | 26.05/28.03 | 0.99748 | 0.99867 | 0.0124 |
| 18 | Vanillin | 10,87 | 151,00 | 136,3-92,2 | Neg | y=446.102×+70934.3 | 0.998 | 0.00696 | 0.00793 | 250-10000 | 48.39/64.23 | 0.99679 | 0.99611 | 0.0280 |

**Table 1S. Method validation parameters of LC-MS/MS Analysis (Yilmaz et al., 2018)**

**a RT: Retention time.**

**b Mother ion(m/z): Molecular ions of the standard compounds (m/z ratio)**

**c R2: Coefficient of determination.**

**d RSD: Relative standard deviation.**

**e LOD/LOQ (µg/L): Limit of detection/quantification.**

**f U (%): percent relative uncertainty at 95% confidence level (k = 2).**

**Figures S. LC chromatograms of the studied *Thymus* extracts**


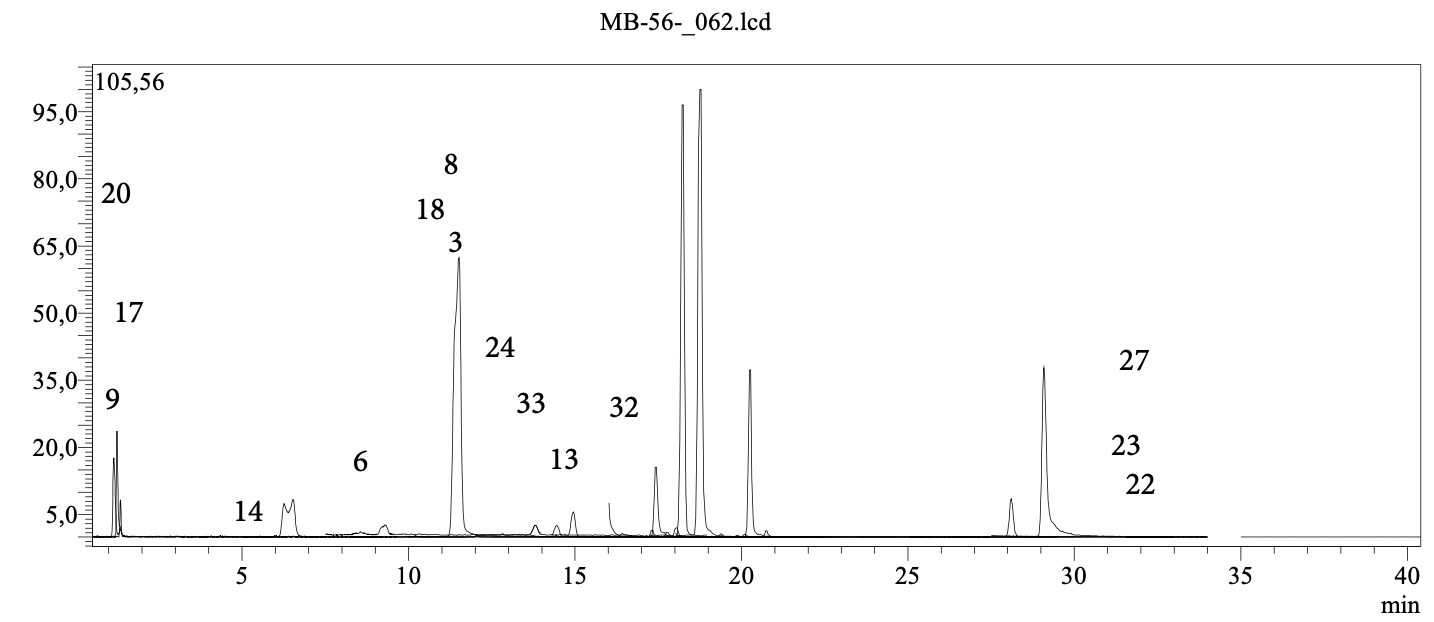


**Figure 1.** LC chromatogram of Tc extract


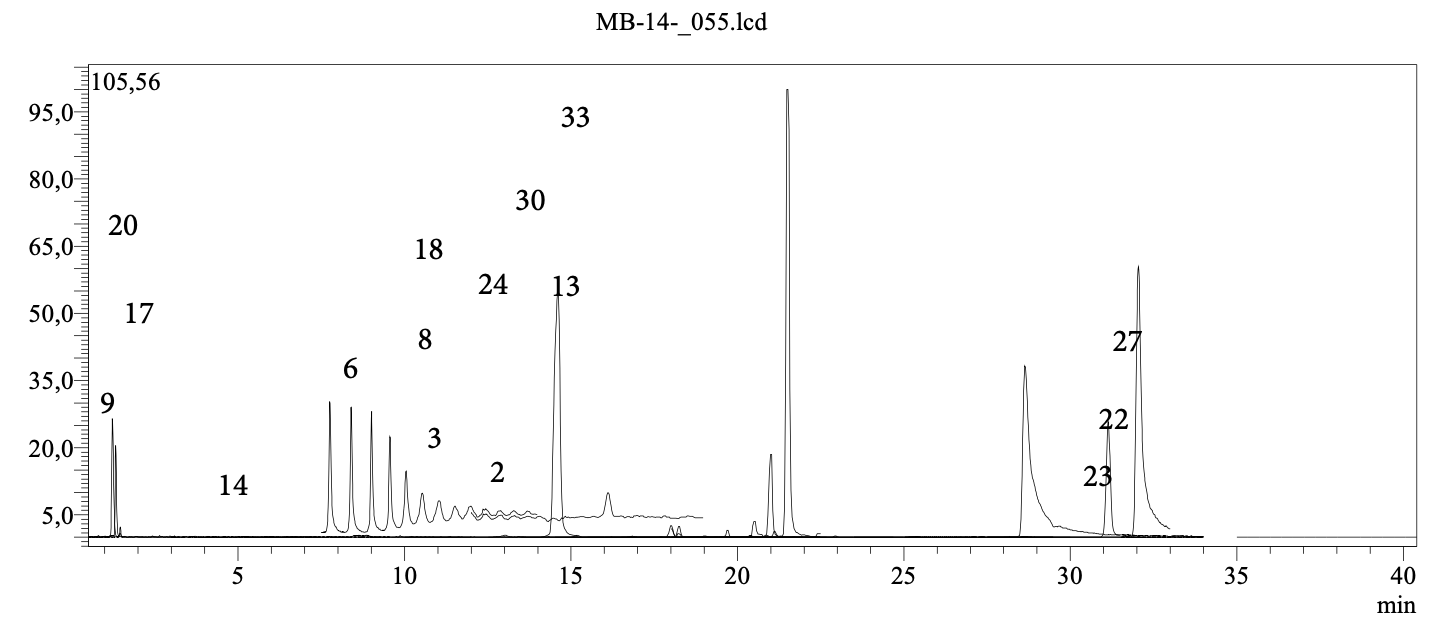


**Figure 2.** LC chromatogram of TprA extract


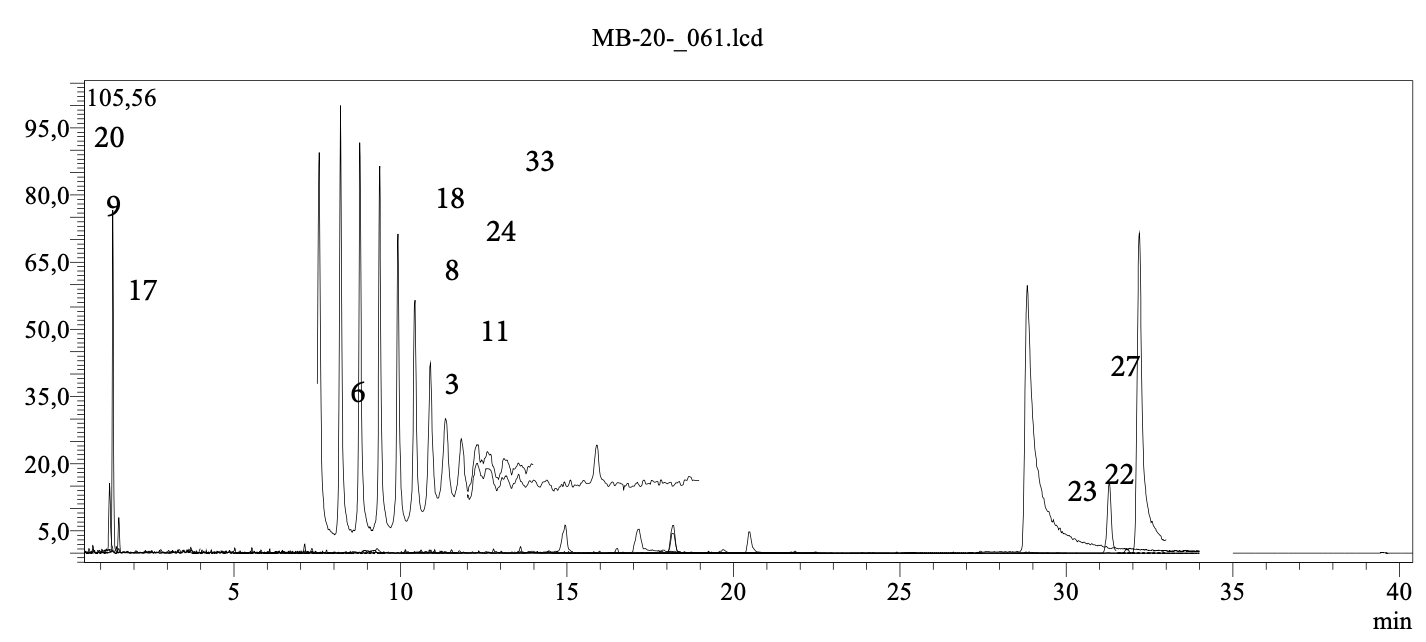


**Figure 3**. LC chromatogram of TprR extract


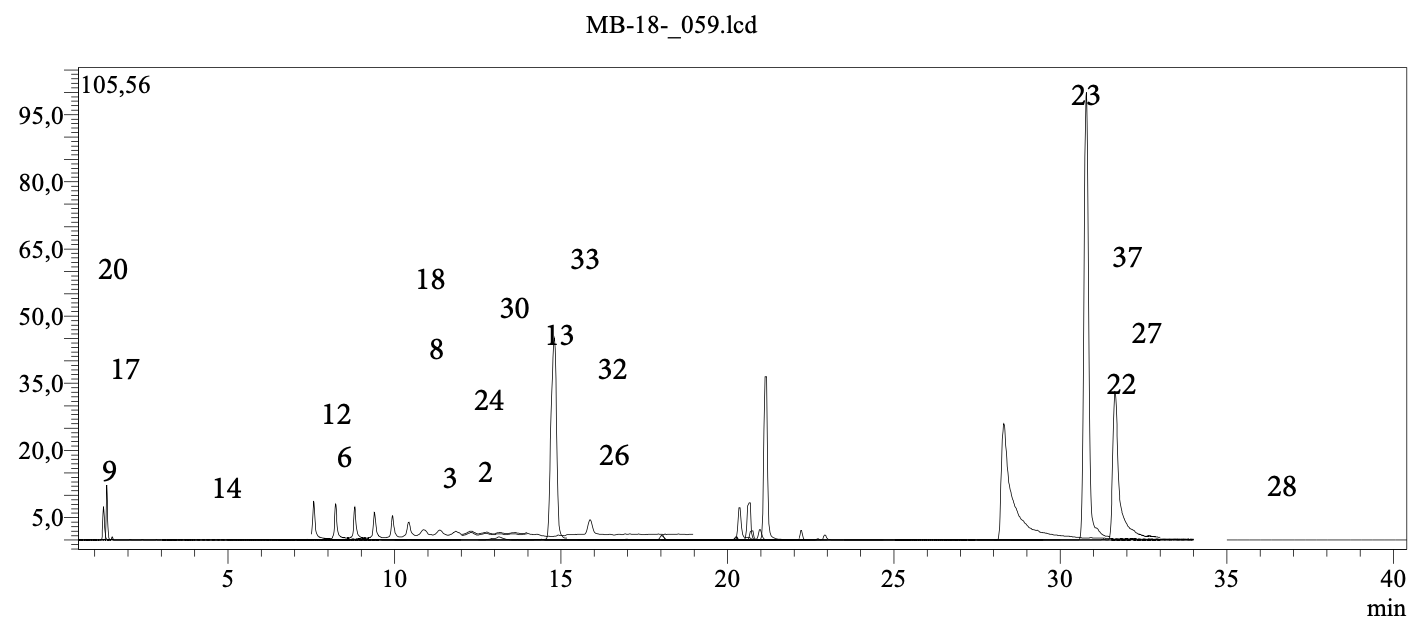


**Figure 4.** LC chromatogram of TpuA extract


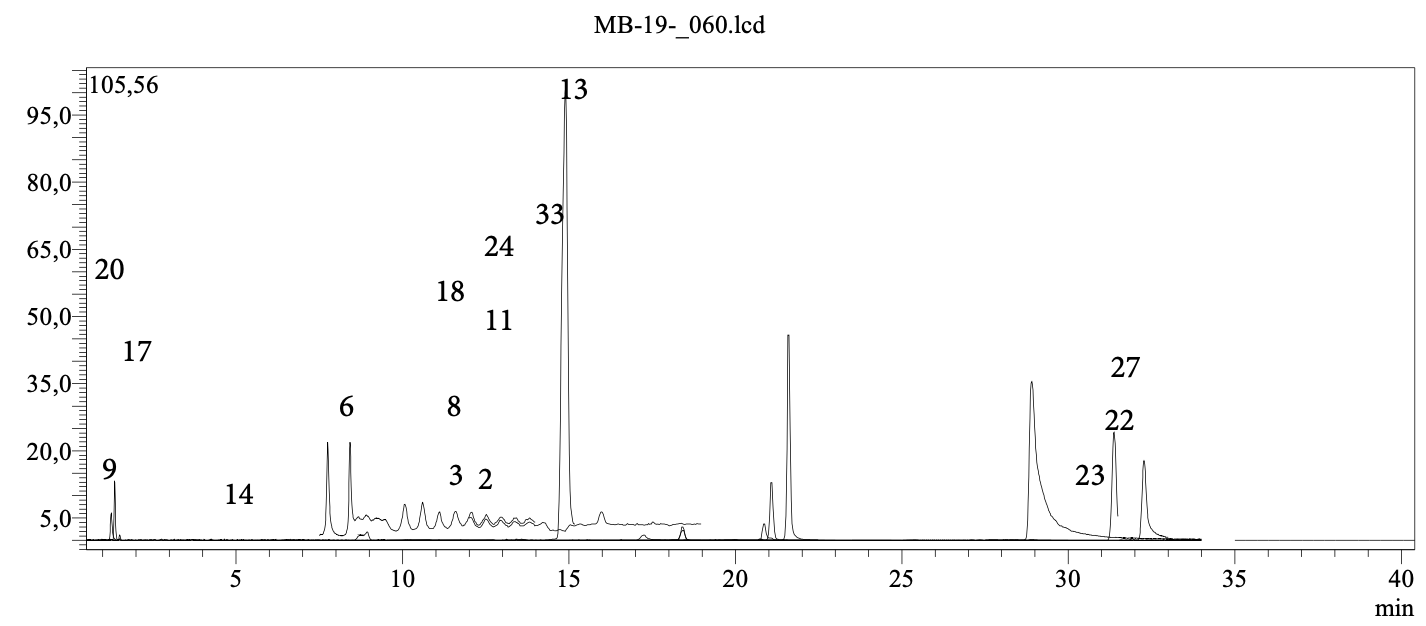


**Figure 5**. LC chromatogram of TpuR extract
